# Quantitative implications of the updated EARL 2019 PET–CT performance standards

**DOI:** 10.1186/s40658-019-0257-8

**Published:** 2019-12-26

**Authors:** Andres Kaalep, Coreline N. Burggraaff, Simone Pieplenbosch, Eline E. Verwer, Terez Sera, Josee Zijlstra, Otto S. Hoekstra, Daniela E. Oprea-Lager, Ronald Boellaard

**Affiliations:** 1 Department of Medical Technology, North Estonia Medical Centre Foundation, J. Sutiste Str 19, Tallinn, 13419 Republic of Estonia; 2 Department of Hematology, Amsterdam UMC, Vrije Universiteit Amsterdam, Cancer Center Amsterdam, Amsterdam, the Netherlands; 3 Department of Radiology and Nuclear Medicine, Amsterdam UMC, Vrije Universiteit Amsterdam, Cancer Center Amsterdam, De Boelelaan 1117, Amsterdam, 1081HV the Netherlands; 4 Department of Nuclear Medicine, University of Szeged, Szeged, Hungary; 5 On behalf of EANM Research Limited (EARL), Vienna, Austria; 6 Department of Nuclear Medicine and Molecular Imaging, University of Groningen, University Medical Centre Groningen, Groningen, the Netherlands

**Keywords:** Performance, Harmonisation, PET, CT, Quantification, EARL accreditation, Standards

## Abstract

**Purpose:**

Recently, updated EARL specifications (EARL2) have been developed and announced. This study aims at investigating the impact of the EARL2 specifications on the quantitative reads of clinical PET–CT studies and testing a method to enable the use of the EARL2 standards whilst still generating quantitative reads compliant with current EARL standards (EARL1).

**Methods:**

Thirteen non-small cell lung cancer **(**NSCLC) and seventeen lymphoma PET–CT studies were used to derive four image datasets—the first dataset complying with EARL1 specifications and the second reconstructed using parameters as described in EARL2. For the third (EARL2F6) and fourth (EARL2F7) dataset in EARL2, respectively, 6 mm and 7 mm Gaussian post-filtering was applied. We compared the results of quantitative metrics (MATV, SUVmax, SUVpeak, SUVmean, TLG, and tumor-to-liver and tumor-to-blood pool ratios) obtained with these 4 datasets in 55 suspected malignant lesions using three commonly used segmentation/volume of interest (VOI) methods (MAX41, A50P, SUV4).

**Results:**

We found that with EARL2 MAX41 VOI method, MATV decreases by 22%, TLG remains unchanged and SUV values increase by 23–30% depending on the specific metric used. The EARL2F7 dataset produced quantitative metrics best aligning with EARL1, with no significant differences between most of the datasets (p>0.05). Different VOI methods performed similarly with regard to SUV metrics but differences in MATV as well as TLG were observed. No significant difference between NSCLC and lymphoma cancer types was observed.

**Conclusions:**

Application of EARL2 standards can result in higher SUVs, reduced MATV and slightly changed TLG values relative to EARL1. Applying a Gaussian filter to PET images reconstructed using EARL2 parameters successfully yielded EARL1 compliant data.

## Introduction

Positron emission tomography (PET) and computed tomography (CT) hybrid imaging (PET–CT) with ^18^F-fluorodeoxyglucose (^18^F-FDG) is widely being used in oncology for diagnosis, staging, restaging and therapy response evaluation due to its ability to measure metabolic changes [1–7]. In addition to visual inspection, quantitative PET data analysis [8] can provide additional benefits such as increased precision and reduced inter-observer variability [9]. Standardised uptake value (SUV) is commonly used to represent the tissue radioactivity concentration normalised to the whole body activity concentration, estimated from injected activity and body weight [10]. However, SUV bias and increased variability can arise from multiple factors [9, 11] and need to be given extra consideration when multicentre data are desired or absolute quantitative measures used [9, 12–15].

Therefore, several scientific societies such as the European Association of Nuclear Medicine (EANM), American College of Radiology (ACR), American Association of Physicists in Medicine (AAPM), Radiological Society of North America (RSNA) and Society of Nuclear Medicine and Molecular Imaging (SNMMI) are promoting standardisation and harmonisation of imaging procedures and practices to reduce variability of quantification in a multicentre setting [16–20]. Results and experience with these programs are described in papers by Scheuermann et al. [19], Sunderland et al. [21] and Kaalep et al. [22].

In 2006, EANM Research Ltd. (EARL) initiative was launched by the EANM to promote multicentre nuclear medicine and research. In 2010, the EARL ^18^F-FDG-PET–CT accreditation program was established to address variability in the quickly growing field of quantitative ^18^F-FDG PET imaging by setting up guidelines and specifications to which the participating sites must adhere. According to these guidelines, an accredited PET–CT system, in addition to other requirements, has to display a SUV bias of ±10% or less and produce contrast recoveries within a specified bandwidth (EARL1 and EARL2), when imaging hot spheres of various sizes within a NEMA NU2–2007 phantom.

The varying performance caused by multiple generations of PET–CT systems (2D, 3D acquisition, time-of-flight (TOF), etc.) and availability of various reconstruction technologies (e.g. resolution recovery/point spread function (PSF) or Bayesian penalised-likelihood reconstruction) pose a particular problem when harmonisation within the community is desired. Multicentre standards should not be based on the least performing systems. They need to fit with the highest, yet common, denominator in systems’ performance.

The specifications for the EARL1 ^18^F-FDG PET–CT accreditation program were developed during a pilot study performed during 2010–2011, involving 12 PET–CT systems. Since then, the performance of PET–CT systems has significantly increased and new acquisition and reconstruction technologies have been introduced [23]. A change in technology may also unfavourably affect patient management when previously used quantitative and visual criteria are applied without adaptation to the new images [24, 25]. The harmonisation of these newer systems would require an update of the current multicentre accreditation standards (EARL1) to accommodate higher recoveries. A phantom study by Kaalep et al. [26] showed that the harmonisation of modern PET–CT systems from different vendors is feasible. Based on this study, the EARL1 specifications have been updated and EARL2 specifications were developed [26].

The introduction of an updated EARL standard changes quantitative PET–CT reads and these changes should be known and/or accounted for. Moreover, for ongoing multicenter studies, it is advisable to continue generating data following the EARL1 standard to assure uniformity of image quantification for the entire multicenter dataset. The latter may be challenging for imaging sites as it could imply that three reconstructions are generated. One reconstruction following locally preferred settings optimised for lesion detectability, another reconstruction following EARL2 and a third reconstruction following EARL1 standards. Therefore, it is of interest to explore if an existing approach based on image filtering [27] can be applied to generate EARL1 compliant data from EARL2 reconstructed images. This would obviate the need to perform a (third) EARL1-compliant reconstruction. Moreover, it would still allow to generate both EARL2- and EARL1-compliant quantitative results to allow comparison of results with historic cohorts. Although in principle the image filtering approach can be applied to the clinically preferred reconstructions to generate either (or both) EARL1- or EARL2-compliant data, the filter settings required would vary from one site to another as locally preferred reconstruction settings are not the same. In a multicenter study, this would require that these filter settings be derived, known and monitored for each system as this method does not generate EARL-compliant images. Yet, deriving EARL1-compliant data from EARL2 reconstructed images is a more standardised condition or procedure and might be more easily reproduced elsewhere.

The primary aim of the current study is to investigate the impact of EARL2 updated accreditation specifications on the quantitative reads of clinical PET–CT studies. A secondary objective is to evaluate the performance of an (existing) approach based on image filtering to generate quantitative reads that are compliant with the EARL1 standards from EARL2-compliant reconstructed PET images.

## Materials and methods

### Selecting post-filtering parameters by phantom experiments

Twenty-one phantom images from a previously described study by Kaalep et al. [26] were investigated. These phantom images served to determine a post-filter which, when applied to an EARL2-compliant dataset, would result in a dataset compliant with the EARL1 standards. The data were collected from 17 EARL accredited scanners from major vendors—3 Philips, 9 Siemens and 5 General Electric systems. The phantom experiments were performed in compliance with EARL Image Quality QC standard operating procedures. A NEMA NU2-2007 body phantom background was filled with a 2 kBq/ml ^18^F-FDG solution and the 10-, 13-, 17-, 22-, 28- and 37-mm spheres with a 20 kBq/ml ^18^F-FDG solution, resulting in a 10:1 sphere to background ratio when scanned for two 5-min per bed positions. TOF, PSF, normalisation, randoms, scatter and attenuation corrections were applied. Reconstructions compliant with EARL1 as well as EARL2 specifications were performed. Reconstructed data were analysed using a semi-automatic tool developed for EARL [16]. Further details regarding the acquisition of the phantom data can be found in the initial study [26].

An additional Gaussian post-filtering with kernel sizes of 5, 6, 7 and 8 mm, respectively, was applied to the EARL2-compliant phantom datasets using in-house post-processing and analysis software ACCURATE [28]. Size-dependent SUVmean and SUVmax recovery coefficients of all resulting datasets were compared with the EARL1 accreditation specifications to determine which filter values provided the largest number of EARL1-compliant results. A dataset was determined to be EARL1 compliant when observed contrast recoveries of SUVmean and SUVmax in all spheres were within EARL1 specifications. This strategy and methodology is equal to the one proposed and evaluated by Lasnon et al. [27] and tested here to see if it could also be applied to derive EARL1-compliant results from EARL2 reconstructed PET data.

### Patient selection and preparation

Thirty patients with non-small cell lung cancer (NSCLC, *n*=13) or lymphoma (*n*=17) were randomly selected from ongoing routine clinical staging or restaging studies with suspected positive lesions. The majority of lymphoma patients were diagnosed with diffuse large B-cell lymphoma. A standard uptake time of 60–75 min was applied to all patients. Further details can be found in Table 1.

**Table 1:** Patient characteristics

Characteristics	Non-small cell lung cancer	Lymphoma	Total
Total no. of patients	13	17	30
Males/females	6 / 7	10 / 7	16 / 14
Weight of patients (mean)	42 – 92 (69.4) kg	61 – 103 (78.9) kg	42 – 103 (74.8) kg
BMI of patients (mean)	18 – 28 (23.3)	19 – 41 (25.7)	18 – 41 (24.7)
Administered activity (mean)	161 – 314 (240) MBq	205 – 347 (274) MBq	161 – 347 (259) MBq
Total no. of analysed lesions	19	36	55
Lesions per patient (mean)	1-3 (1.5)	1-3 (2.1)	1-3 (1.8)

### Acquisition and reconstruction parameters

30 patient scans performed on two EARL-accredited Philips Ingenuity PET–CT TOF systems during a period of 2.5 years (from 10.03.2016 to 10.09.2018) were selected for further analysis within this study. For each patient study, two PET reconstructions were performed—first, using the EARL1-approved reconstruction parameters resulting in contrast recoveries within EARL1 accreditation specifications [29], and second, using the EARL2-compliant reconstruction parameters proposed by Kaalep et al [26]. Two additional image datasets were generated from the EARL2-reconstructed images by applying Gaussian filters of 6.0 mm (EAR2F6) and 7.0 mm (EAR2F7), respectively. Main parameters for the four PET image datasets used in this study are listed in Table 2.

**Table 2:** Main reconstruction parameters of the four investigated image datasets

Reconstruction	Pixel spacing (mm)	Slice thickness (mm)	Reconstruction method	Post-filter width (mm)	Resolution recovery
EARL1	4	4	BLOB-OS-TF	N/A	OFF
EARL2	4	4	BLOB-OS-TF	N/A	ON
EARL2F6	4	4	BLOB-OS-TF	6.0	ON
EARL2F7	4	4	BLOB-OS-TF	7.0	ON

### Lesion selection, segmentation and analysis

Lesions suspected to be malignant were identified by the author (AK) and confirmed by a certified nuclear medicine physician with 10 years of experience reading PET–CT images (DO). Physiological ^18^F-FDG uptake (i.e. within brain, left ventricle, kidneys, urinary bladder) not related to the primary disease was excluded. A maximum of 3 lesions per patient were selected to avoid over-representation of any single patient, yielding a total of 55 lesions. VOIs were segmented semi-automatically using a region-growing method with the following thresholds:

1. SUV ≥ 4.0 (SUV4).
2. 41% of SUVmax (41MAX).
3. 50% of SUVpeak with adaptation to local tumor-to-background contrast, so-called adapted 50% of SUVpeak (A50P) [30]. SUVpeak is defined as the average uptake in a 1.2-cm-diameter VOI positioned such to yield the highest value across all tumor voxels (also referred to as highest peak) [31].

Additionally for each patient, liver and blood pool VOIs were created, and SUVmax, SUVpeak and SUVmean calculated for EARL1, EARL2 and EARL2F7 reconstructions. For assessing liver uptake, we positioned a 3-cm-diameter spherical VOI in the right upper lobe of the (healthy) liver, as suggested by PERCIST [31]. For the blood pool uptake, a 1.5-cm spherical VOI was positioned in the lumen of the ascending aorta.

For each combination of lesion, reconstruction and VOI threshold, the following quantitative metrics were calculated: metabolic active tumor volume (MATV), SUVmean, SUVmax, SUVpeak and total lesion glycolysis (TLG) [30]. Moreover, we derived tumor-to-liver ratios using SUVmax of both lesion and liver as well as SUVmax of the lesion and SUVmean of the liver. Brief descriptions of the used VOI methods and quantitative metrics are given in Table 3.

**Table 3:** Descriptions of used VOI methods and quantitative metrics

VOI methods	Description
A50P	Region-growing-based VOI using 50% of SUVpeak with adaptation to local tumor-to-background contrast [32, 30]
SUV4	Region-growing-based VOI using a threshold of SUV equal to 4
MAX41	Region-growing-based VOI using a threshold equal to 41% of SUVmax
Quantitative metrics	Description
MATV	Volume of a lesion segmented using A50P, SUV4 or MAX41 method in mL
SUV_mean_	Ratio of image-derived average radioactivity concentration within a region of interest and the estimated whole body concentration of the injected radioactivity, normalised to bodyweight
SUV_max_	Ratio of image-derived maximum (single pixel) radioactivity concentration within a region of interest and the whole body concentration of the injected radioactivity, normalised to bodyweight
SUV_peak_	Ratio of image-derived average radioactivity concentration within a 12-mm-diameter spherical volume (taking into account fractional voxels) within the region of interest, positioned to yield the highest uptake across all tumor voxel locations, and the whole body concentration of the injected radioactivity, normalised to bodyweight [30, 31]
TLG	Total lesion glycolysis equal to the MATV times SUVmean

### Statistical analyses

Median relative differences of quantitative metrics determined from EARL2, EARL2F6 and EARL2F7 reconstructions and corresponding values from the EARL1 reconstruction were reported along with interquartile ranges. Mann–Whitney *U* test and Wilcoxon signed-rank test were used to evaluate the statistical significance of the paired and non-paired data, respectively.

## Results

### Phantom data

**Fig. 1:**
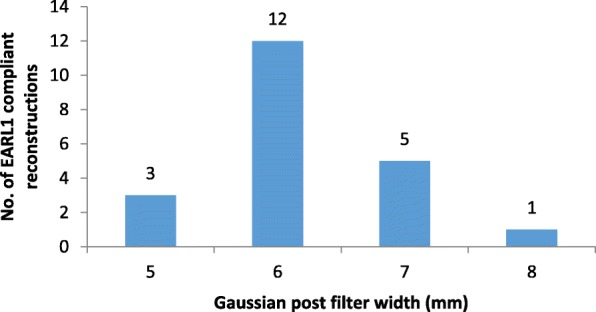
Histogram of Gaussian post-filter values resulting in EARL1-compliant reconstructions of NEMA NU2-2007 body phantom data acquired in accordance with EARL guidelines for Image Quality QC standard operating procedures

**Fig. 2:**
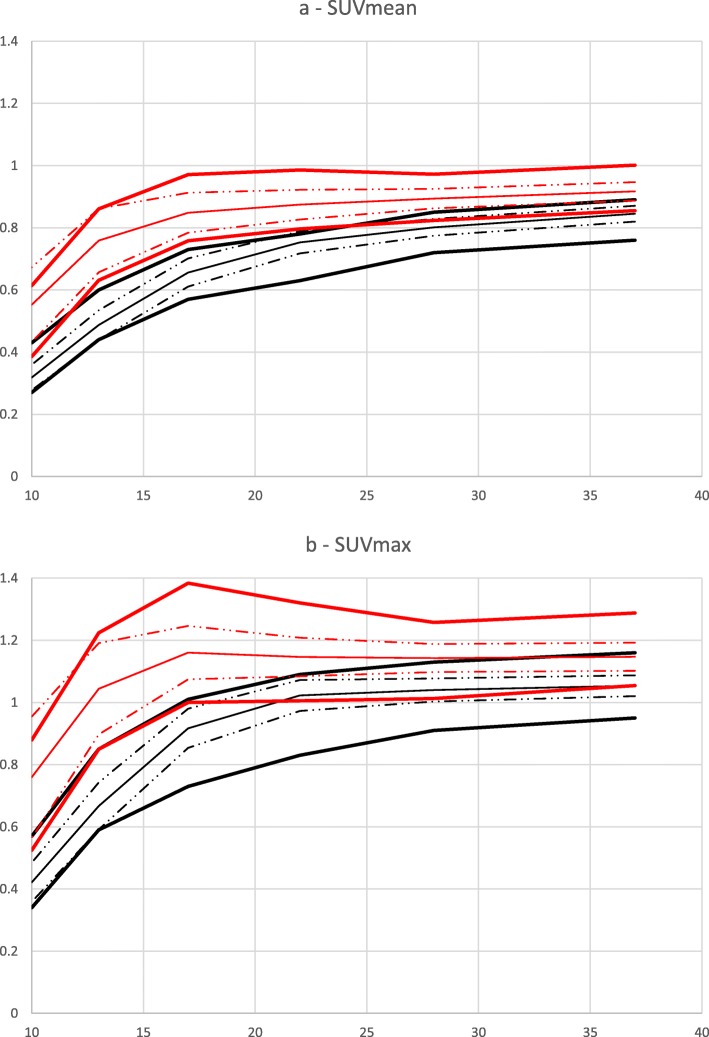
Recovery coefficient values relative to sphere size determined from NEMA NU2-2007 body phantom data acquired in accordance with EARL guidelines for Image Quality QC standard operating procedures. **a** SUVmean of EARL2 and EARL2F6 data. **b** SUVmax of EARL2 and EARL2F6 data. Average measured EARL1 SUV data—solid black line, average measured EARL2F6 SUV data—solid red line current, standard deviation from average measured EARL1 SUV data—black dash dot dot line; standard deviation from average measured EARL2F6 SUV data—red dash dot dot line; EARL standard—bold black lines, prospective future EARL standard—bold red lines

Post-filtering values of 6 and 7 mm resulted in the highest number of EARL2 reconstructions (across all phantom scans) conforming to EARL1 specifications for both SUVmean and SUVmax (Fig. 1) and were selected for further analysis and testing using clinical datasets. As an example, Fig. 2 demonstrates the investigated systems’ contrast recovery curves before and after the application of an additional 6-mm FWHM Gaussian post-filter.

### Clinical data

**Fig. 3:**
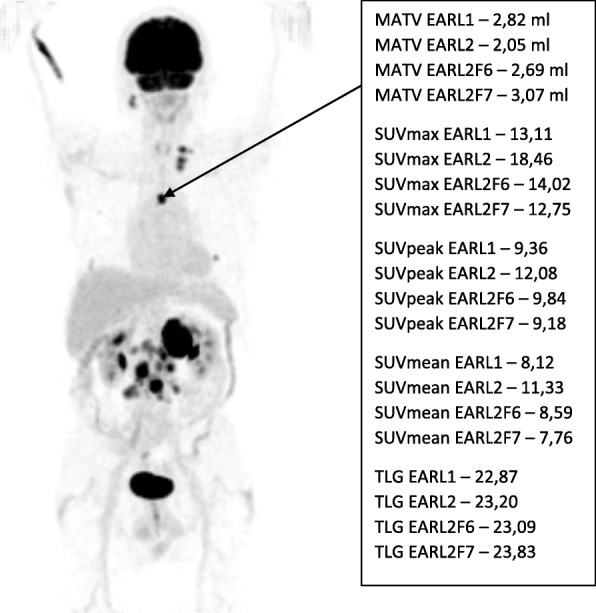
Typical example image of a lymphoma patient’s ^18^F-FDG PET–CT scan. Coronal maximum intensity projection image is presented with an arrow pointing to a suspected malignant lesion along with the corresponding MATV, SUVmax, SUVpeak, SUVmean and TLG quantitative metrics determined using EARL1, EARL2, EARL2F6 and EARL2F7 reconstructions

**Fig. 4:**
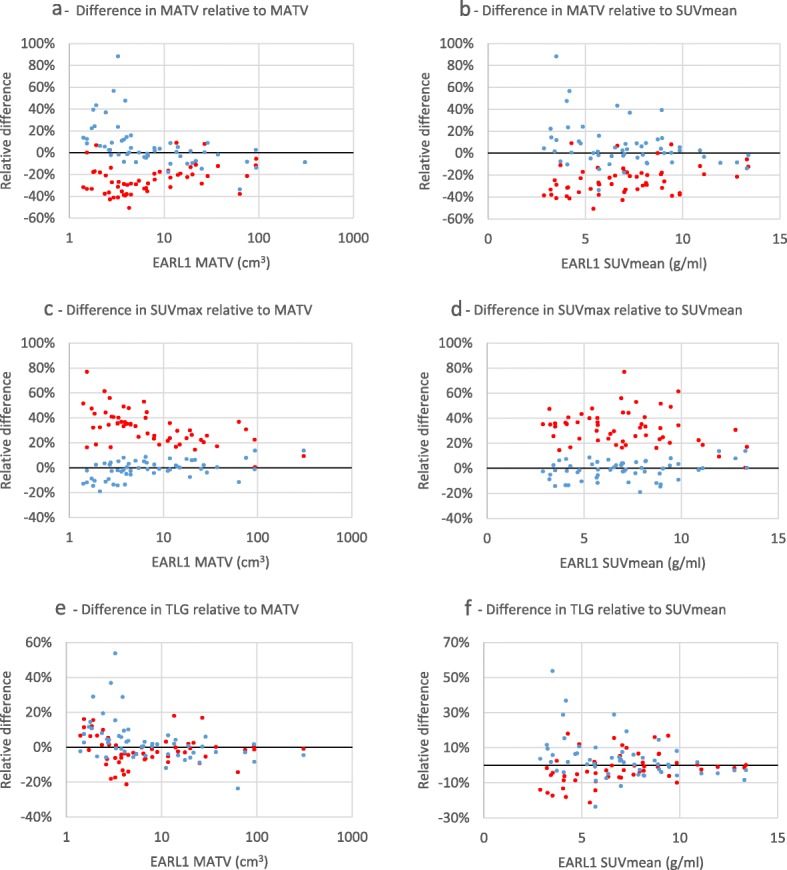
Relative differences of 41MAX VOI method EARL2 and EARL2F7 reconstructions’ MATV, SUVmax and TLG metrics compared to respective values from EARL1 reconstruction, presented as functions of EARL1 MATV and SUVmean. EARL2 reconstruction—red markers; EARL2F7 reconstruction—blue markers

Figure 3 illustrates an example of a malignant lesion and the associated quantitative metrics calculated. We found that EARL2 SUV data were higher than EARL1 data (*p*<0.001), the relative median difference ranging from 23% for SUVpeak to 25% for SUVmean and 30% for SUVmax (Table 4, Fig. 4c, d). The relative difference in SUV between the two datasets increased with decreasing MATV (*p*<0.001) with a median difference of 36%, 39% and 25% (SUVmax, SUVmean and SUVpeak, respectively) for small (< 10 ml) lesions and 22%, 21% and 15% (SUVmax, SUVmean and SUVpeak, respectively) for large (≥ 10 ml) lesions (Fig. 4c), with no significant dependence on SUVmean, i.e. lesion contrast (SUVmax, *p*=0.162; SUVmean, *p*=0.225; SUVpeak, *p*=0.178) (Fig. 4d). SUV data from the filtered dataset EARL2F7 aligned best with those obtained using EARL1 reconstruction within -1% for SUVmax (interquartile range 9.5), -1% for SUVmean (interquartile range 9.4) and +2% for SUVmax (interquartile range 6.4).

**Table 4:** MAX41 VOI method relative median differences (%) of MATV, SUV metrics and TLG to corresponding values of EARL1 reconstruction along with 25th and 75th percentiles. Values marked with * indicate that the difference is statistically significant (*p* < 0.05)

Cancer type	Reconstruction	MATV	SUVmax	SUVpeak	SUVmean	TLG	Tumor SUVmax/liver SUVmax	Tumor SUVmax/liver SUVmean
Lung cancer	EARL_V2	-22* (-31/-15)	30* (22/35)	23* (14/25)	25* (18/36)	-1 (-5/2)	18* (12/22)	29* (23/35)
	EARL_V2F6	0 (-6/2)	3* (1/6)	5* (4/7)	3* (2/6)	2 (-1/4)	N/A	N/A
	EARL_V2F7	9 (1/14)	-3 (-8/0)	1 (-2/3)	-2 (-7/0)	4 (-2/8)	6* (1/9)	-3 (-8/0)
Lymphoma	EARL_V2	-28* (-36/-19)	35* (22/41)	24* (17/29)	37* (23/42)	-3* (-8/1)	26* (14/30)	35* (22/41)
	EARL_V2F6	-7* (-12/-2)	6* (2/13)	6* (3/11)	6* (0/12)	0 (-4/4)	N/A	N/A
	EARL_V2F7	0 (-5/7)	0 (-4/4)	2 (-2/4)	0 (-4/4)	0 (-5/5)	6* (1/8)	0 (-4/4)
Both combined	EARL_V2	-27* (-33/-18)	33* (22/40)	23* (16/28)	34* (22/40)	-2* (-7/2)	22* (14/29)	34* (23/39)
	EARL_V2F6	-5* (-11/1)	5* (1/11)	5* (3/9)	5* (2/9)	1 (-3/4)	N/A	N/A
	EARL_V2F7	2 (-4/11)	-1 (-6/4)	2* (-2/4)	-1 (-5/4)	2 (-4/7)	6* (1/9)	-1 (-6/3)

MATV seen on the EARL2 was on average 27% smaller (*p*<0.001) when compared to EARL1 (Table 4, Fig. 4a, b), while the difference reduced to a statistically non-significant +2% (*p*=0.547) when EARL2F7 and EARL1 were compared. Results for the A50P and SUV4 VOI methods can be found in the supplemental data (Additional file 5: Table S1 and Table S2). The relative difference in EARL2 and EARL1 MATV values was found to be dependent on the underlying MATV values (*p*<0.001) with an average difference of -31% for small (< 10 ml) lesions and -19% for large (≥ 10 ml) lesions (Fig. 4a). Significant MATV dependence (*p*<0.001) remained when EARL2F7 and EARL1 were compared, with a median difference of +6% for small (< 10 ml) lesions and -8% for large (≥ 10 ml) lesions. A significant dependence of underlying SUV levels (*p*=0.033) was found in MATV differences of EARL2 and EARL1, where lesions with SUVmean ≤ 7.0 demonstrated a median difference of -29% while lesions with SUVmean > 7.0 demonstrated a median difference of -20%. Differences in MATV of EARL2F7 to EARL1 were independent (*p*=0.076) on underlying SUV levels (Fig. 4b).

We found a statistically significant (*p*=0.005) difference of -2% in median TLG values of EARL1 and EARL2 while no statistically significant difference in TLG derived from EARL1 and EARL2F7 (*p*=0.744) or EARL1 and EARL2F6 (*p*=0.815).

**Fig. 5:**
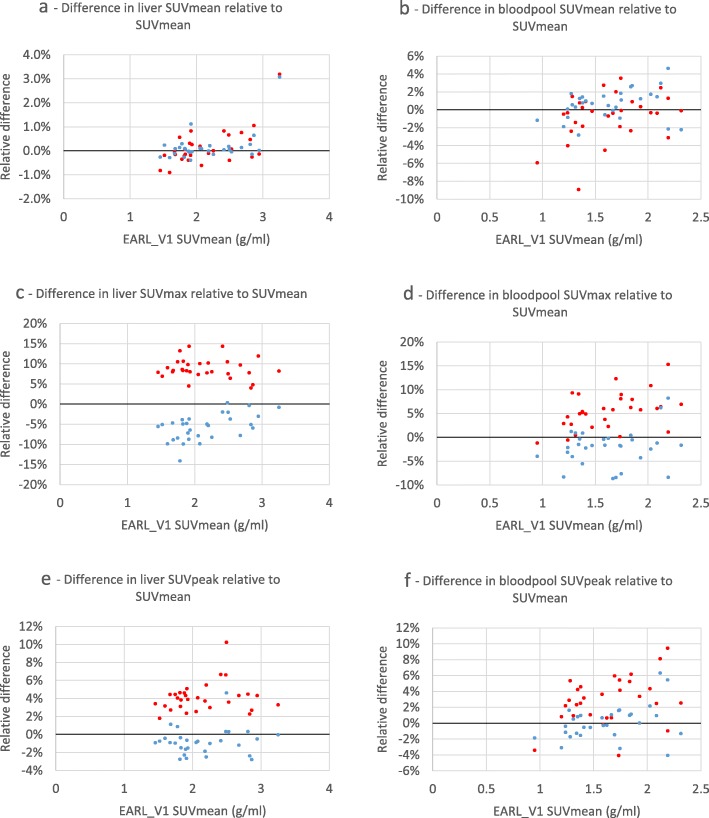
Relative differences of 41MAX VOI method EARL2 and EARL2F7 reconstructions’ liver and blood pool SUVmean, SUVmax and SUVpeak metrics compared to respective values from EARL1 reconstruction, presented as functions of EARL1 SUVmean. EARL2 reconstruction—red markers, EARL2F7 reconstruction—blue markers

**Fig. 6:**
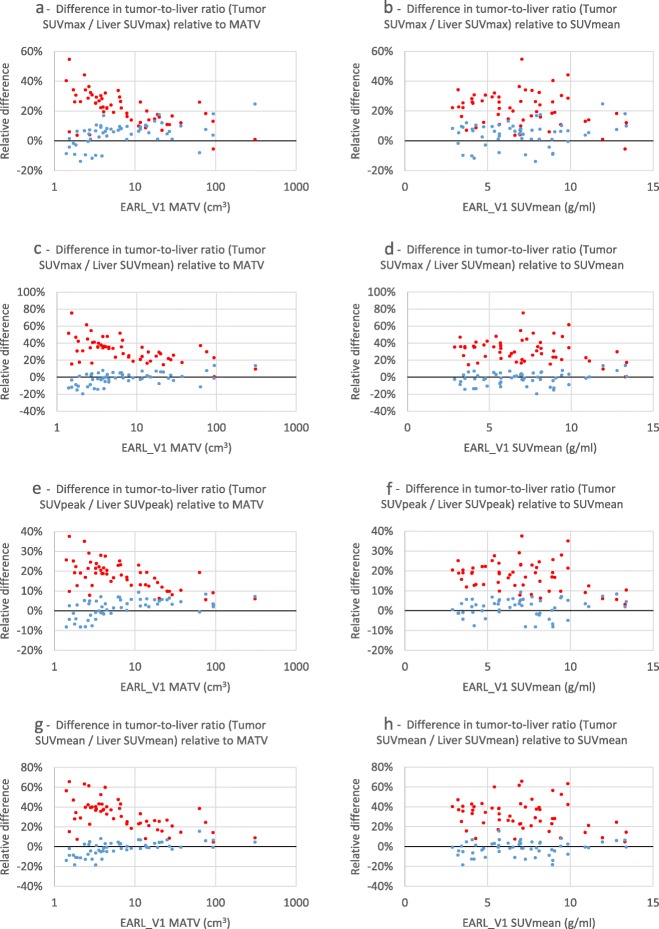
Relative differences of 41MAX VOI method EARL2 and EARL2F7 reconstructions’ tumor-to-liver ratios compared to respective values from EARL1 reconstruction, presented as functions of EARL1 MATV and SUVmean. **a** Tumor SUVmax to liver SUVmax ratio relative to MATV; **b** tumor SUVmax to liver SUVmax ratio relative to SUVmean; **c** tumor SUVmax to liver SUVmean ratio relative to MATV; **d** tumor SUVmax to liver SUVmean ratio relative to SUVmean; **e** tumor SUVpeak to liver SUVpeak ratio relative to MATV; **f** tumor SUVpeak to liver SUVpeak ratio relative to SUVmean; **g** tumor SUVmean to liver SUVmean ratio relative to MATV; **h** tumor SUVmean to liver SUVmean ratio relative to SUVmean; EARL2 reconstruction—red markers, EARL2F7 reconstruction—blue markers

Figure 5 demonstrates the differences in SUV in liver and blood pool VOIs between EARL1 and EARL2 and EARL2F7 reconstructions. The largest difference can be seen with SUVmax while SUVmean demonstrates the smallest change. Figure 6 visualises tumor-to-liver ratio’s calculated using various combinations of SUV metrics. Additional results comparing EARL2 and EARL2F7 reconstructions can be found in the supplemental data (Additional file 2: Figure S2; Additional file 3: Figure S3; Additional file 4: Figure S4).

**Fig. 7:**
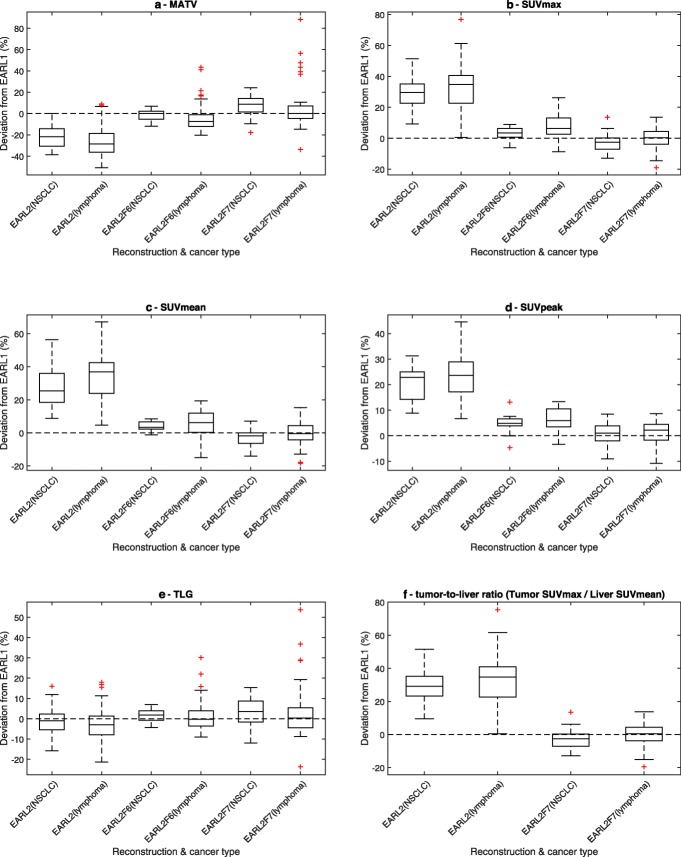
Comparison of relative differences of MATV (**a**), SUVmax (**b**), SUVmean (**c**), SUVpeak (**d**), TLG (**e**) and tumor-to-liver ratio (tumor SUVmax relative to liver SUVmean) (**f**) metrics between EARL1 and EARL2, EARL2F6, and EARL2F7 reconstructions, respectively, using 41MAX VOI method. Results obtained from lung cancer and lymphoma patients are presented separately. Central line of the box is the median, edges of the box are the 25th and 75th percentiles, the whiskers extend to either of the most extreme data points, which are not considered outliers or 1.5 times interquartile range. The outliers are marked using plus signs

Tumor-to-liver ratio was significantly higher for EARL2 when either SUVmax (*p*<0.001) or SUVmean (*p*<0.001) was used to measure liver SUV (Table 4). For EARL2F7, the difference with EARL1 data was still statistically significant (*p*<0.001) when SUVmax was used to measure liver SUV and non-significant (*p*=0.344) when liver SUVmean was used. All investigated metrics changed similarly between EARL2, EARL2F6 and EARL2F7 reconstructions regardless of cancer type (Fig. 7). SUVmax and SUVpeak metric changes remained similar while SUVmean, MATV and TLG behaviour differed based on the VOI method used (Additional file 1: Figure S1). These differences between VOI methods were eliminated in the EARL2F7 reconstruction (Additional file 1: Figure S1).

## Discussion

Application of the updated EARL2 standards will affect quantitative reads. Yet, an update of the current EARL1 standards is required to cope with new PET–CT technologies providing enhanced lesion detectability. In this paper, we studied the impact of using EARL2 standards on the quantitative reads of NSCLC and lymphoma ^18^F-FDG PET–CT studies as compared to the EARL1 standards. An (existing) image filtering approach that enables the use of updated EARL2-compliant reconstruction, whilst still generating EARL1-compliant quantitative reads, was derived and tested. The latter is important to allow comparison of data during ongoing studies and/or for comparison with historical cohorts.

As previously shown in the phantom study by Kaalep et al. [26], EARL2-compliant reconstruction resulted in a significant increase in contrast recovery and higher SUVs. The current study confirms these findings for clinical data (Table 4) and demonstrates that overall trends (Table 4, Fig. 7) were similar for both lung cancer and lymphoma patients despite different lesion sizes and tracer uptake levels (or SUV). This suggests that the results of the current study could be universally applicable regardless of underlying disease type but this should be further investigated in future studies covering a wider range of patients and conditions.

Increase in contrast in EARL2-compliant reconstructions is similar for the most commonly used metrics SUVmax and SUVpeak applying all investigated VOI methods (Additional file 1: Figure S1) and is independent of the lesion contrast (SUVmean). However, the results demonstrate a dependence on lesion volume where smaller lesions show a larger increase in contrast. The overall increased contrast recovery explains the wider and generally more preferred use of PSF reconstructions [23].

EARL2-compliant reconstructions result in a significant reduction in MATV values when MAX41 VOI method is used. Small (< 10 ml) lesions demonstrate a relatively higher decrease in volume than larger (≥ 10 ml) lesions. At the same time, smaller lesions demonstrate larger increase in all SUV metrics which could be explained by the improved resolution and reduced spill-out effect caused by the PSF reconstruction. Post-filtering of the EARL2F6 and EARL2F7 reconstructions mimics the spill-out effect as the image is blurred by the filter resulting in loss of resolution and dispersion of measured activity in a larger volume. These results should not be transferred to other VOI methods as the PSF reconstruction affects different lesion segmentation methods differently.

TLG did not change significantly among the four investigated reconstructions. This may have been expected since while the lesion contrast (SUVmean) increases, MATV decreases proportionally, resulting in a reduced change in the product of the two. This reconstruction-independent behaviour of TLG could potentially make it a good metric for generating consistent quantitative measurements from both EARL1 and EARL2 standards without further image modification. This is in agreement with Armstrong et al., who suggested that TLG may be less sensitive to reconstruction methods compared with either SUVmax or SUVpeak [33]. TLG could also reduce the uncertainty of quantitative measurements of PET–CT scans that are performed on a system with unknown recovery coefficients. These results should not be transferred to SUV4 VOI methods as the PSF reconstruction affects the different lesion segmentations and, therefore, the corresponding TLG values also.

EARL1-compliant quantitative results can be generated from EARL2-reconstructed data using a simple filter. This approach has been applied and validated before by Lasnon et al., specifically for PSF reconstruction on a Siemens Biograph PET–CT system using the so-called EQ.PET approach [27]. The method could, however, be considered limited due to lack of similar solutions by other major vendors. Moreover, the appropriate filter for each PET–CT system is not reported in the DICOM header and thereby the method cannot be applied offline or by 3rd party software. In our paper, we identified (from phantom experiments) and verified the appropriate Gaussian filter setting to use EARL2-compliant reconstructions, while enabling the generation of EARL1 quantitative results. Also, it is important for the verification of new datasets to be equivalent to historical cohorts. In this way, data can be easily generated to conform to both EARL1 and EARL2 standards without a need for additional reconstructions. For ongoing studies, however, it is recommended to keep adherence to the EARL1 standard and optionally adding EARL2-compliant reconstructions to gain understanding of quantitative implications when transitioning to the new standard.

It has been demonstrated by Kuhnert et al*.* that the increase in SUV, brought about by the use of PSF reconstruction, remains significant even after normalisation to the liver [24]. In our study, we also found that normalising lesion uptake to either liver SUVmax or liver SUVmean resulted in significantly increased tumor-to-liver ratios and confirm the results of Kuhnert et al that normalising to liver uptake does not mitigate the effect of using different reconstructions on lesion uptake assessments. Apart from harmonising quantitative ^18^F-FDG PET–CT reads, it has been reported that also visual assessment, e.g. Deauville scoring of PET–CT lymphoma studies, may be affected by a change in (reconstruction) technology and could affect patient management [25]. The observed changes (increases) in tumor-to-liver ratios for EARL2- versus EARL1-compliant data suggest that the use of EARL2 standards will result in overall higher Deauville scores, similarly as recently found by Ly et al. [34]. Therefore, it is not recommended to change standards and/or use new technologies without properly (re-)defining interpretation criteria. Hence, use of EARL2-compliant reconstruction in combination with generating a second filtered dataset could be helpful in recalibrating these criteria for studies performed to conform EARL1 performance standards to those obtained using updated performance standards. However, it should be noted that although this filtering approach has been shown to yield EARL1-compliant results and may facilitate these type of studies, this has not yet been demonstrated in this study nor was the aim.

## Conclusions

Multicentre clinical trials require standardisation of quantitative results to be usable and exchangeable. This can be challenging as new acquisition and reconstruction technologies emerge and enable great benefits in image quality, but at the same time cause the quantitative performance to diverge.

In this paper we studied the impact of using updated EARL2 standards on the quantitative reads of NSCLC and lymphoma ^18^F-FDG PET–CT studies as compared to the EARL1 standards. In general, the new EARL2 guidelines resulted in higher SUVs, smaller MATV and similar TLG values. A 7-mm FWHM Gaussian filter was shown to convert EARL2-compliant PET data to EARL1-compliant images. This facilitates the generation of both new and existing EARL-compliant quantitative reads from a single EARL2-compliant image reconstruction.

## Supplementary information

**Additional file 1: Figure S1.** Comparison of relative differences of MATV (**a**), SUVmax (**b**), SUVmean (**c**), SUVpeak (**d**), TLG (**e**) and tumor-to-liver ratio (tumor SUVmax relative to liver SUVmean) (**f**) metrics between EARL1 and EARL2, EARL2F6, and EARL2F7 reconstructions, respectively, using different VOI methods. Central line of the box is the median, edges of the box are the 25th and 75th percentiles, the whiskers extend to either of the most extreme data points, which are not considered outliers or 1.5 times interquartile range. The outliers are marked using plus signs.

**Additional file 2: Figure S2.** Relative differences of A50P VOI method EARL2 and EARL_V2F7 reconstructions’ MATV, SUVmax and TLG metrics compared to respective values from EARL1 reconstruction, presented as functions of EARL1 MATV and SUVmean. EARL2 reconstruction—red markers, EARL2F7 reconstruction—blue markers.

**Additional file 3: Figure S3.** Relative differences of SUV4 VOI method EARL2 and EARL2F7 reconstructions’ MATV, SUVmax and TLG metrics compared to respective values from EARL1 reconstruction, presented as functions of EARL1 MATV and SUVmean. EARL2 reconstruction—red markers, EARL2F7 reconstruction—blue markers.

**Additional file 4: Figure S4.** Relative differences of 41MAX VOI method EARL2 and EARL2F7 reconstructions’ tumor-to-bloodpool ratio compared to respective values from EARL1 reconstruction, presented as functions of EARL1 MATV and SUVmean. a tumor SUVmax to bloodpool SUVmax ratio relative to MATV; b tumor SUVmax to bloodpool SUVmax ratio relative to SUVmean; c tumor SUVmax to bloodpool SUVmean ratio relative to MATV; d tumor SUVmax to bloodpool SUVmean ratio relative to SUVmean; e tumor SUVpeak to bloodpool SUVpeak ratio relative to MATV; f tumor SUVpeak to bloodpool SUVpeak ratio relative to SUVmean; g tumor SUVmean to bloodpool SUVmean ratio relative to MATV; h tumor SUVmean to bloodpool SUVmean ratio relative to SUVmean; EARL2 reconstruction—red markers, EARL2F7 reconstruction—blue markers.

**Additional file 5: Table S1.** A50P VOI method relative median differences (%) of MATV, SUV metrics and TLG to corresponding values of EARL1 reconstruction along with corresponding interquartile ranges. Values marked with * indicate that the difference is statistically significant (p < 0.05). **Table S2.** SUV4 VOI method relative median differences (%) of MATV, SUV metrics and TLG to corresponding values of EARL1 reconstruction along with corresponding interquartile ranges. Values marked with * indicate that the difference is statistically significant (p < 0.05).

## Data Availability

Data will be made available at https://zenodo.org/.

## Abbreviations

^18^F-FDG: ^18^F-fluorodeoxyglucose
A50P: Region-growing-based VOI using 50% of SUVpeak with adaptation to local tumor-to-background contrast
AAPM: American Association of Physicists in Medicine
ACR: American College of Radiology
BMI: Body mass index
CT: computed tomography
EANM: European Association of Nuclear Medicine
EARL: EANM Research Ltd.
MATV: Volume of a lesion segmented using A50P, SUV4 or MAX41 method in mL
MAX41: Region-growing-based VOI using a threshold equal to 41% of SUVmax
NSCLC: Non-small cell lung cancer
PET: Positron emission tomography
PSF: Point spread function
RSNA: Radiological Society of North America
SNMMI: Society of Nuclear Medicine and Molecular Imaging
SUV: Standardised uptake value
SUV4: Region-growing-based VOI using a threshold of SUV equal to 4
SUV_max_: Ratio of image-derived maximum (single pixel) radioactivity concentration within a region of interest and the whole body concentration of the injected radioactivity, normalised to bodyweight
SUV_mean_: Ratio of image-derived average radioactivity concentration within a region of interest and the estimated whole body concentration of the injected radioactivity, normalised to bodyweight
SUV_peak_: Ratio of image-derived average radioactivity concentration within a 12-mm-diameter spherical volume within the region of interest, positioned to yield the highest uptake, and the whole body concentration of the injected radioactivity, normalised to bodyweight
TLG: Total lesion glycolysis equal to the MATV times SUVmean
TOF: Time of flight
VOI: Volume of interest
