# Supplementary material for: Quantitative implications of the updated EARL 2019 PET–CT performance standards
Source: EJNMMI Phys. 2019 Dec 26;6:28. doi: 10.1186/s40658-019-0257-8 (PMC6933045; doi:10.1186/s40658-019-0257-8)
Supplement: Supplementary file 5 — Additional file 5: Table S1. A50P VOI method relative median differences (%) of MATV, SUV metrics and TLG to corresponding values of EARL1 reconstruction along with corresponding interquartile ranges. Values marked with * indicate that the difference is statistically significant (p < 0.05). Table S2. SUV4 VOI method relative median differences (%) of MATV, SUV metrics and TLG to corresponding values of EARL1 reconstruction along with corresponding interquartile ranges. Values marked with * indicate that the difference is statistically significant (p < 0.05). [file 40658_2019_257_MOESM5_ESM.docx]

# Additional file

**Table S1.** A50P VOI method relative median differences (%) of MATV, SUV metrics and TLG to corresponding values of EARL1 reconstruction along with corresponding interquartile ranges. Values marked with * indicate that the difference is statistically significant (p < 0.05)

| **Cancer type** | **Reconstruction** | **MATV** | **SUVmax** | **SUVpeak** | **SUVmean** | **TLG** |
| --- | --- | --- | --- | --- | --- | --- |
| Lung cancer | EARL2 | -9* (10) | 30* (13) | 24* (13) | 22* (14) | 8* (6) |
|  | EARL2F6 | -3 (9) | 3* (6) | 5* (3) | 4* (3) | 2 (7) |
|  | EARL2F7 | 2 (14) | -3* (8) | 1 (6) | -1 (5) | 1 (8) |
| Lymphoma | EARL2 | -15* (10) | 35* (19) | 24* (10) | 28* (19) | 7* (8) |
|  | EARL2F6 | -4 (10) | 6* (11) | 6* (7) | 5* (8) | 1 (7) |
|  | EARL2F7 | -1 (21) | 0* (8) | 2 (6) | -1 (11) | 0 (9) |
| Both combined | EARL2 | -14* (13) | 34* (17) | 24* (10) | 25* (15) | 7* (7) |
|  | EARL2F6 | -4* (10) | 5* (10) | 5* (5) | 4* (4) | 2 (7) |
|  | EARL2F7 | 0 (18) | -1 (8) | 2* (6) | -1 (7) | 0 (9) |

**Table S2.** SUV4 VOI method relative median differences (%) of MATV, SUV metrics and TLG to corresponding values of EARL1 reconstruction along with corresponding interquartile ranges. Values marked with * indicate that the difference is statistically significant (p < 0.05)

| **Cancer type** | **Reconstruction** | **MATV** | **SUVmax** | **SUVpeak** | **SUVmean** | **TLG** |
| --- | --- | --- | --- | --- | --- | --- |
| Lung cancer | EARL2 | 10 (63) | 33* (10) | 23* (11) | 13* (8) | 27* (70) |
|  | EARL2F6 | 3* (7) | 4* (6) | 5* (3) | 2* (2) | 5* (6) |
|  | EARL2F7 | 1* (10) | -3* (8) | 1 (6) | -1* (3) | 0 (10) |
| Lymphoma | EARL2 | 3 (18) | 36* (21) | 25* (10) | 14* (8) | 22* (24) |
|  | EARL2F6 | 4* (7) | 6* (11) | 6* (7) | 2* (5) | 7* (10) |
|  | EARL2F7 | 3* (5) | 0* (11) | 2 (6) | -1* (5) | 1 (7) |
| Both combined | EARL2 | 4* (28) | 35* (19) | 24* (10) | 14* (6) | 23* (33) |
|  | EARL2F6 | 3* (7) | 5* (9) | 5* (5) | 2* (3) | 6* (8) |
|  | EARL2F7 | 3* (5) | -1 (10) | 2* (6) | -1* (4) | 1* (7) |
