# Supplementary material for: Quantitative implications of the updated EARL 2019 PET–CT performance standards
Source: EJNMMI Phys. 2019 Dec 26;6:28. doi: 10.1186/s40658-019-0257-8 (PMC6933045; doi:10.1186/s40658-019-0257-8)
Supplement: Supplementary file 2 [file 40658_2019_257_MOESM2_ESM.docx]

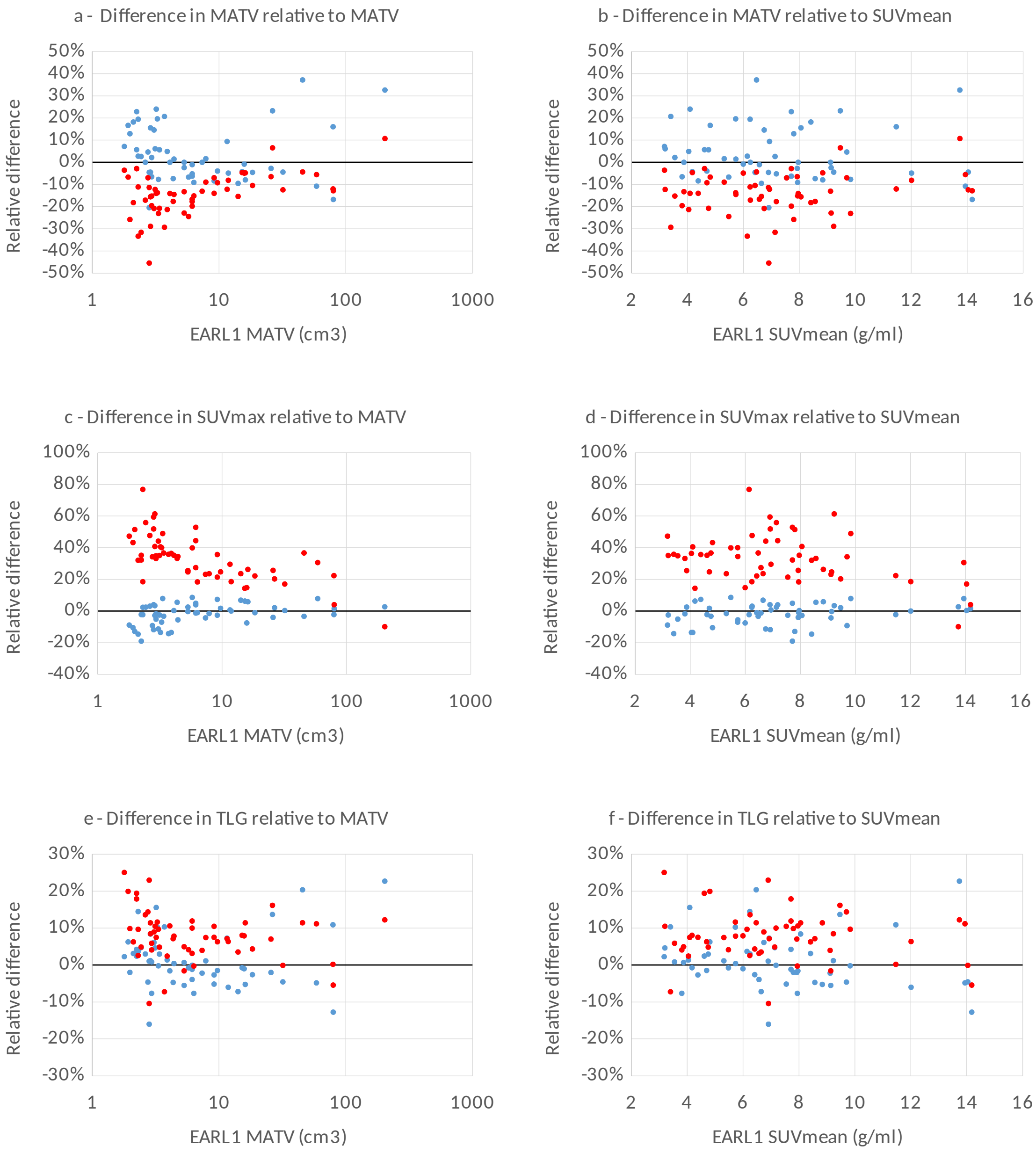
a - Difference in MATV relative to MATV
Relative difference
50%
40%
30%
20%
10%
0%
-10%
-20%
-30%
-40%
-50%
1
10
100
1000
EARL1 MATV (cm3)
b - Difference in MATV relative to SUVmean
Relative difference
2
4
6
8
10
12
14
16
EARL1 SUVmean (g/ml)
c - Difference in SUVmax relative to MATV
Relative difference
100%
80%
60%
40%
20%
0%
-20%
-40%
EARL1 MATV (cm3)
d - Difference in SUVmax relative to SUVmean
Relative difference
EARL1 SUVmean (g/ml)
e - Difference in TLG relative to MATV
Relative difference
30%
20%
10%
0%
-10%
-20%
-30%
EARL1 MATV (cm3)
f - Difference in TLG relative to SUVmean
Relative difference
EARL1 SUVmean (g/ml)
