# Supplementary material for: Quantitative implications of the updated EARL 2019 PET–CT performance standards
Source: EJNMMI Phys. 2019 Dec 26;6:28. doi: 10.1186/s40658-019-0257-8 (PMC6933045; doi:10.1186/s40658-019-0257-8)
Supplement: Supplementary file 3 [file 40658_2019_257_MOESM3_ESM.docx]

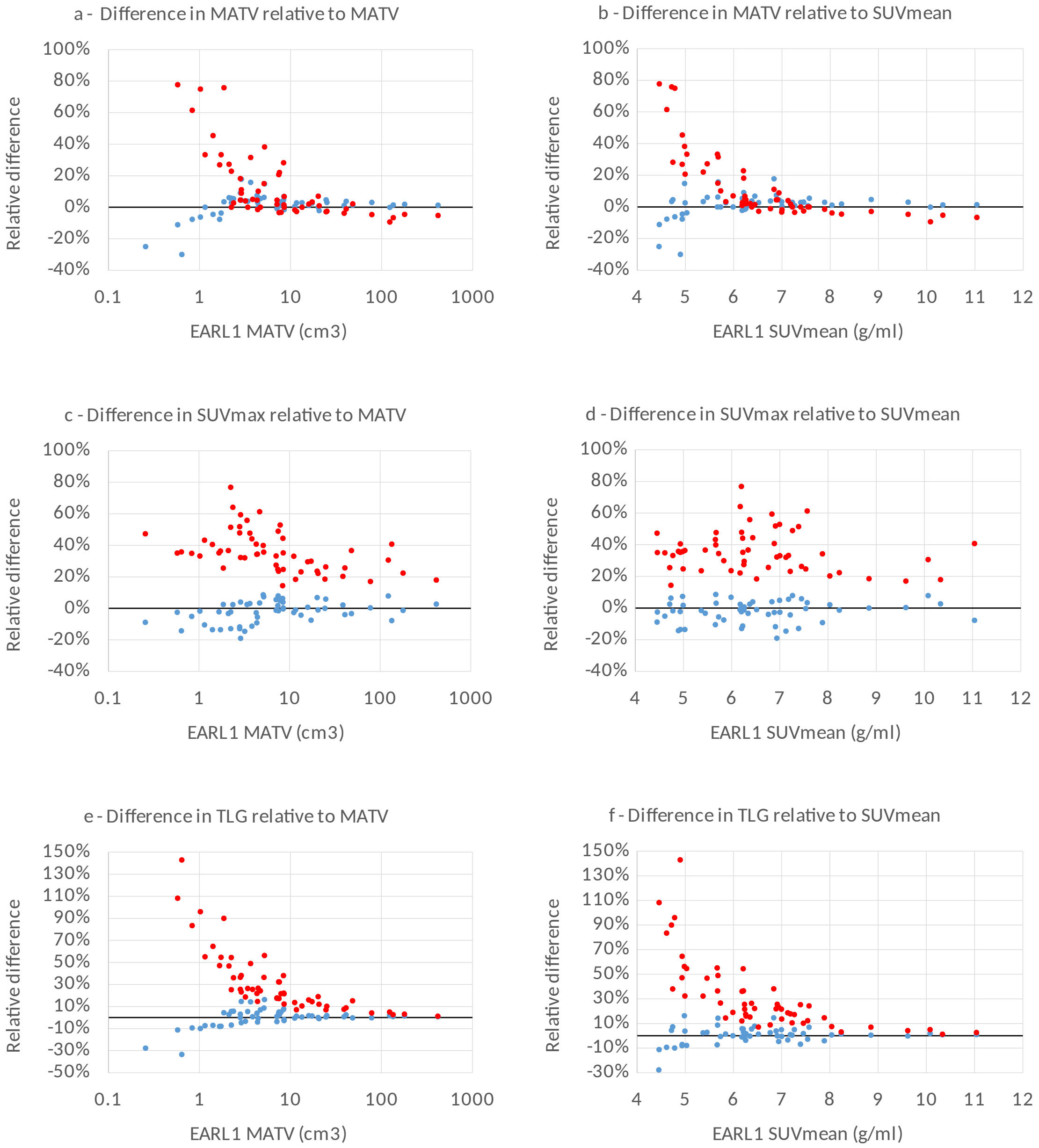
a - Difference in MATV relative to MATV
Relative difference
100%
80%
60%
40%
20%
0%
-20%
-40%
0.1
1
10
100
1000
EARL1 MATV (cm3)
b - Difference in MATV relative to SUVmean
Relative difference
100%
80%
60%
40%
20%
0%
-20%
-40%
4
5
6
7
8
9
10
11
12
EARL1 SUVmean (g/ml)
c - Difference in SUVmax relative to MATV
Relative difference
100%
80%
60%
40%
20%
0%
-20%
-40%
0.1
1
10
100
1000
EARL1 MATV (cm3)
d - Difference in SUVmax relative to SUVmean
Relative difference
100%
80%
60%
40%
20%
0%
-20%
-40%
4
5
6
7
8
9
10
11
12
EARL1 SUVmean (g/ml)
e - Difference in TLG relative to MATV
Relative difference
150%
130%
110%
90%
70%
50%
30%
10%
-10%
-30%
-50%
0.1
1
10
100
1000
EARL1 MATV (cm3)
f - Difference in TLG relative to SUVmean
Relative difference
150%
130%
110%
90%
70%
50%
30%
10%
-10%
-30%
4
5
6
7
8
9
10
11
12
EARL1 SUVmean (g/ml)
