# Supplementary material for: Quantitative implications of the updated EARL 2019 PET–CT performance standards
Source: EJNMMI Phys. 2019 Dec 26;6:28. doi: 10.1186/s40658-019-0257-8 (PMC6933045; doi:10.1186/s40658-019-0257-8)

a - Difference in tumor-to-bloodpool ratio (tumor SUVmax / bloodpool SUVmax) relative to MATV

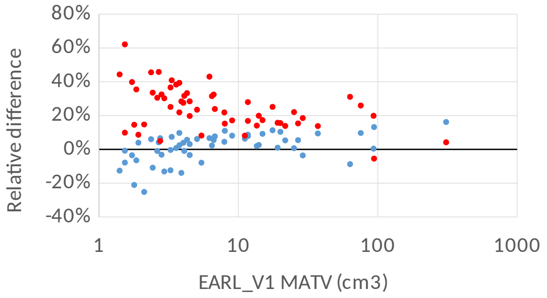

b - Difference in tumor-to-bloodpool ratio (tumor SUVmax / bloodpool SUVmax) relative to SUVmean

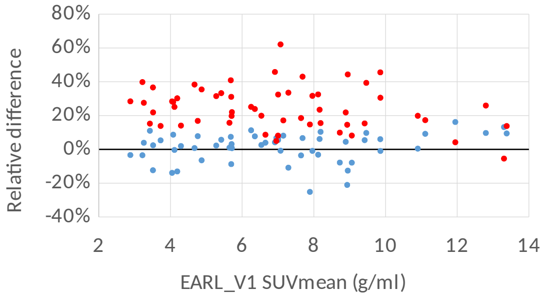

c - Difference in tumor-to-bloodpool ratio (tumor SUVmax / bloodpool SUVmean) relative to MATV

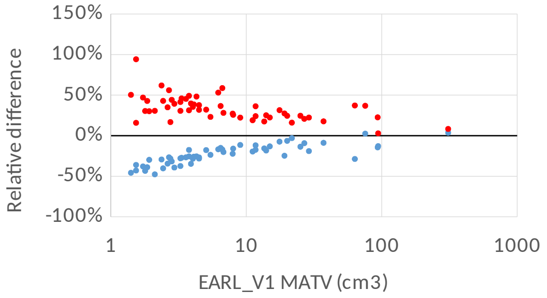

d - Difference in tumor-to-bloodpool ratio (tumor SUVmax / bloodpool SUVmean) relative to SUVmean

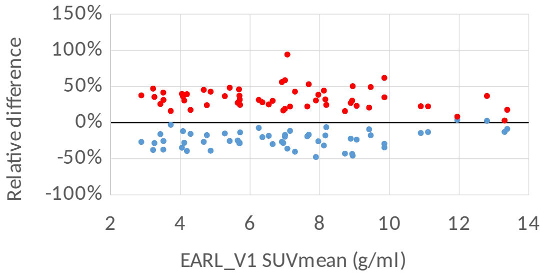

e - Difference in tumor-to-bloodpool ratio (tumor SUVpeak / bloodpool SUVpeak) relative to MATV

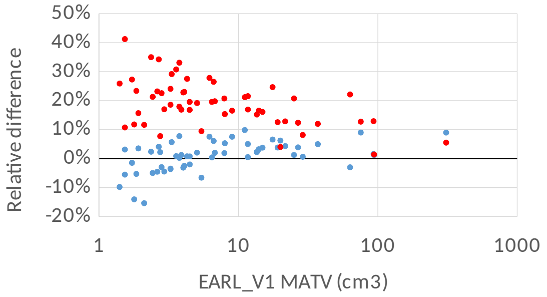

f - Difference in tumor-to-bloodpool ratio (tumor SUVpeak / bloodpool SUVpeak) relative to SUVmean

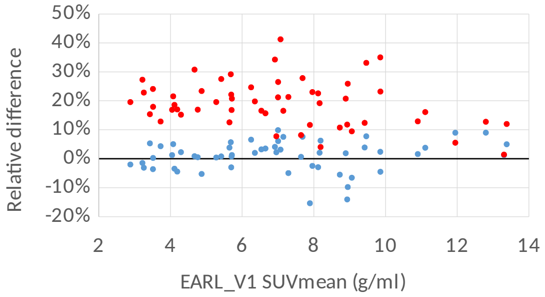

g - Difference in tumor-to-bloodpool ratio (tumor SUVmean / bloodpool SUVmean) relative to MATV

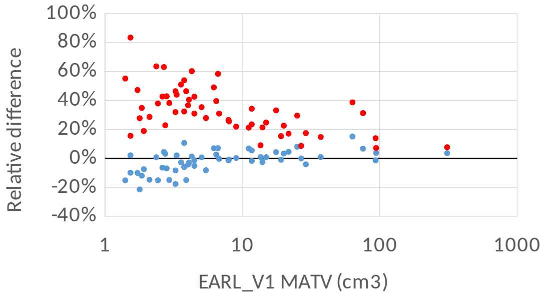

h - Difference in tumor-to-bloodpool ratio (tumor SUVmean / bloodpool SUVmean) relative to SUVmean

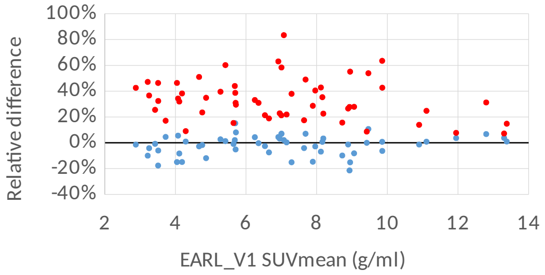

Supplement: Supplementary file 4 [file 40658_2019_257_MOESM4_ESM.docx]
